# Carcinoid tumors outside the abdomen

**DOI:** 10.1002/cam4.5564

**Published:** 2022-12-22

**Authors:** Kenna Koehler, Wade T. Iams

**Affiliations:** ^1^ Department of Medicine, Division of Hematology‐Oncology Vanderbilt University Medical Center Nashville Tennessee USA; ^2^ Vanderbilt‐Ingram Cancer Center Nashville Tennessee USA

**Keywords:** atypical carcinoid, bronchial carcinoid, thymic carcinoid, typical carcinoid

## Abstract

Neuroendocrine tumors (NETs) are epithelial malignancies that can arise from multiple tissues. Gastrointestinal (GI) NETs are the most common; in this review of extra‐abdominal carcinoid tumors, we focus our discussion on bronchial and thymic carcinoid tumors. Bronchial carcinoid tumors comprise a quarter of all NETs and less than 2% of all lung cancers. Thymic carcinoid tumors are extremely rare, accounting for 5% of thymic tumors. Both bronchial and thymic carcinoid tumors are histologically classified as either typical or atypical based on their mitotic rate (less than 2 or 2–10 mitoses per 10 high‐powered fields (HPF), respectively). Both bronchial and thymic carcinoids can present with symptoms of obstruction and potentially carcinoid syndrome. The gold standard of management of bronchial and thymic carcinoid tumors is surgical resection. For patients with advanced disease, first‐line systemic therapy is generally somatostatin analog monotherapy with octreotide or lanreotide. In patients with refractory disease, therapy generally involves peptide receptor radioligand therapy, everolimus, or cytotoxic chemotherapy. There are ongoing, prospective trials comparing the mainstays of systemic therapy for these patients, as well as ongoing evaluations of immune checkpoint inhibitors and multi‐kinase inhibitors. Prognosis for both bronchial and thymic carcinoid tumors depends on histologic grade, local versus invasive disease, and extent of metastases. Herein we provide a summary of the pathophysiologic and clinical background, the current state of the field in diagnosis and management, and note of key ongoing prospective trials for patients with bronchial and thymic carcinoid tumors.

## Graphical abstract

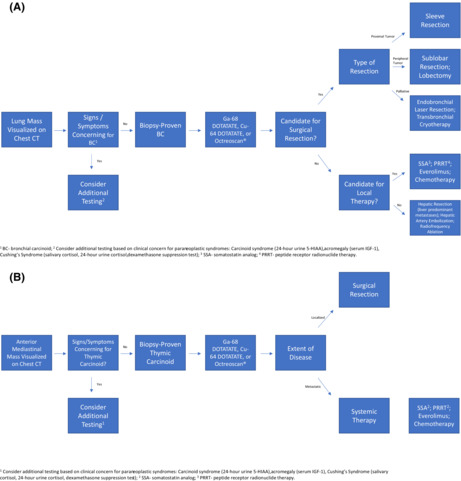

## 1 BACKGROUND

Neuroendocrine tumors (NETs) are epithelial malignancies that arise from Kulchitsky and enterochromaffin‐like cells, which are located in many tissues throughout the body, including the pancreas, small intestine, lung, thymus, skin, and thyroid.^1^ Gastrointestinal (GI) NETs are the most common. While NETs are quite rare, their incidence is rising, likely owing to better diagnostic awareness. NETs have the ability to secrete a variety of bioactive amines, making them a heterogenous group of tumors that range from indolent to aggressive.^2^, ^3^ Bronchial and thymic carcinoid tumors are uncommon NETs arising from the thorax, comprising about a quarter of all NETs.^4^

### 1.1 Bronchial carcinoid

**FIGURE 1:**
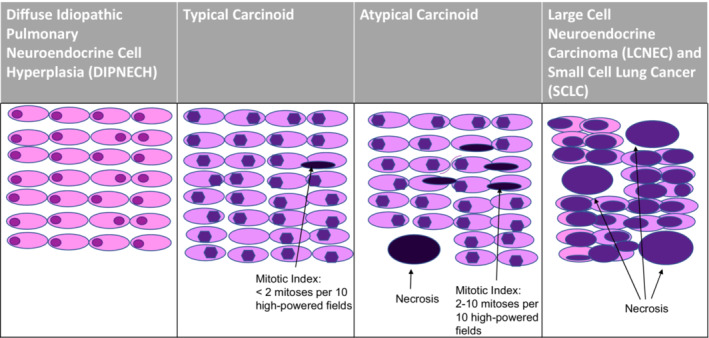
Histologic spectrum of neuroendocrine tumors

Bronchial carcinoid (BC) tumors comprise less than 2% of all lung cancers.^2^ The 2015 World Health Organization (WHO) classification groups pulmonary NETs into typical and atypical carcinoid tumors, small‐cell lung cancer (SCLC), and large‐cell neuroendocrine carcinoma (LCNEC), largely based on mitotic rate.^5^ Histologically, typical carcinoids (TCs) contain cytologically bland cells with few mitotic figures (less than 2 per 10 high powered fields (HPF)) and no necrosis, whereas atypical carcinoids (ACs) contain 2–10 mitoses per 10 HPF and/or necrosis (Figure 1).^6^, ^7^ More indolent neuroendocrine tumors are described as diffuse idiopathic pulmonary neuroendocrine cell hyperplasia (DIPNECH), a pre‐invasive hyperplasia of pulmonary neuroendocrine cells.^5^ BCs fall between the pre‐invasive DIPNECH and more invasive SCLC and LCNEC.^8^ TCs are slow growing, well‐differentiated, low‐grade neoplasms that uncommonly metastasize outside of the thorax.^9^, ^10^ ACs are faster growing, intermediate grade neoplasms that can disseminate early in the clinical course.^9^, ^10^

There is a lack of data regarding risk factors for development of BCs. Some historical studies suggested smoking as a risk factor; however, evidence is weak compared to other types of lung cancer.^11^, ^12^ Multiple endocrine neoplasia types 1 and 4 and familial carcinoids are rare but can predispose to the development of BCs.^13^ Most bronchial carcinoids occur sporadically.

### 1.2 Thymic carcinoid

Thymic carcinoid tumors are far less common than BCs and account for about 5% of all thymic tumors.^14^ They can often be mistaken for thymomas, and, in fact, were not designated their own separate diagnostic entity until 1972.^15^ Thymic carcinoid tumors are also classified as either typical and atypical based on the same histologic parameters as BCs.^14^, ^16^ Similar to BCs, little data exists concerning risk factors for developing thymic carcinoid tumors.

## 2 PRESENTATION

### 2.1 Bronchial carcinoid

While most carcinoid tumors are diagnosed incidentally by imaging obtained for unrelated indications, bronchial carcinoid tumors most commonly present with symptoms of obstruction. Many BCs originate in the proximal airways, and patients can present with chest pain, dyspnea, cough, or wheezing.^17^ BCs are hypervascular and can present with hemoptysis as well. Additionally, there are reports that BCs can present with Cushing syndrome.^18^

### 2.2 Thymic carcinoid

Presenting symptoms of thymic carcinoids are often related to anterior mediastinal compression and include superior vena cava syndrome, dyspnea, chest pain, and hoarseness.^16^ As with BCs, thymic carcinoids have been reported to cause Cushing syndrome.^16^ Rarely, thymic carcinoid can secrete growth hormone.^19^

### 2.3 Carcinoid syndrome

Rarely, BC and thymic carcinoid can present with carcinoid syndrome (CS).^20^, ^21^ CS is a paraneoplastic syndrome that can cause secretory diarrhea, cutaneous flushing, bronchospasm, palpitations, telangiectasias, and cardiac valvular lesions (so called “carcinoid heart” syndrome).^20^ When CS is present, patients with BCs typically have more severe flushing compared to those with GI NETs. CS is caused by secretion of serotonin, histamine, kallikrein, and prostaglandins, and occurs most commonly in GI NETs with metastases to the liver, however, CS can occur in patients with BC or thymic carcinoid tumors as well.^20^ In these patients, CS occurs more often with metastatic disease rather than localized disease.^21^ One study estimated the frequency of CS to be 8% in both localized and regional BC and 15% in advanced BC.^21^ The rate of CS in thymic carcinoid is much more rare.^22^

Table 1 describes the structural and secretory symptoms that are associated with the clinical presentation of BCs and thymic carcinoid tumors.

**TABLE 1:** Extra‐abdominal carcinoid patient presentation

	Bronchial carcinoid	Thymic carcinoid
Structural symptoms	Chest pain Dyspnea Cough Wheezing Hemoptysis Weight loss	Superior vena cava syndrome Dyspnea Hoarseness Chest pain Weight loss
Secretory symptoms	Carcinoid syndrome Cushing's syndrome Acromegaly	Carcinoid syndrome Cushing's syndrome Acromegaly

## 3 DIAGNOSIS AND STAGING

A contrasted chest computed tomography (CT) scan is the preferred imaging modality to assess tumor size, location, and lymphadenopathy in the diagnosis of BC or thymic carcinoid tumor.^23^, ^24^ There are no well‐established tumor markers to detect BCs or thymic carcinoids. Serum chromogranin A (CgA) has been linked to NETs; however, it is more likely to be elevated in patients with GI NETs rather than BCs or thymic carcinoids, thus serum and urine markers are not routinely recommended.^25^ If there is concern about carcinoid syndrome at presentation, clinicians can consider 24‐hour urinary excretion of 5‐HIAA; however, this is most sensitive for abdominal NETs.^26^, ^27^ Clinicians can also consider testing for Cushing's syndrome (late night salivary cortisol, 24‐hour urine cortisol, and/or dexamethasone suppression test) or acromegaly (serum IGF‐1).^28^, ^29^ Diagnosis is most often confirmed via bronchoscopic biopsy for lung carcinoids, as BCs are typically centrally located, and thymic biopsy for thymic carcinoids. In order to reduce the risk of bleeding at the time of biopsy, diluted epinephrine is often administered before and after sampling.

Once a diagnosis of BC (either TC or AC) or thymic carcinoid tumor is confirmed, functional imaging should be obtained. Most BCs and thymic carcinoid tumors express somatostatin receptors (SSTRs), and can be readily visualized on gallium Ga‐68 DOTATATE or copper Cu‐64 DOTATATE positron emission tomography (PET) imaging, or on the older Octreoscan®.^30^ These imaging modalities are useful for assessing for metastases, and they are predictive of response to one of the somatostatin analogs (SSAs).^24^, ^30^ Typically, surveillance imaging is completed with conventional CT imaging, and somatostatin receptor imaging modalities are deployed when results of conventional CT are ambiguous.^30^

BCs are staged using the same American Joint Committee on Cancer (AJCC) 8th edition tumor, lymph node, metastasis (TNM) staging system as other lung carcinomas. Typical BCs tend to present at a lower stage compared to ACs.^31^ Similarly, thymic carcinoids are staged using the same TNM staging system as other thymic carcinomas.^32^

## 4 MANAGEMENT OF LOCALIZED DISEASE

### 4.1 Bronchial carcinoid

**FIGURE 2:**
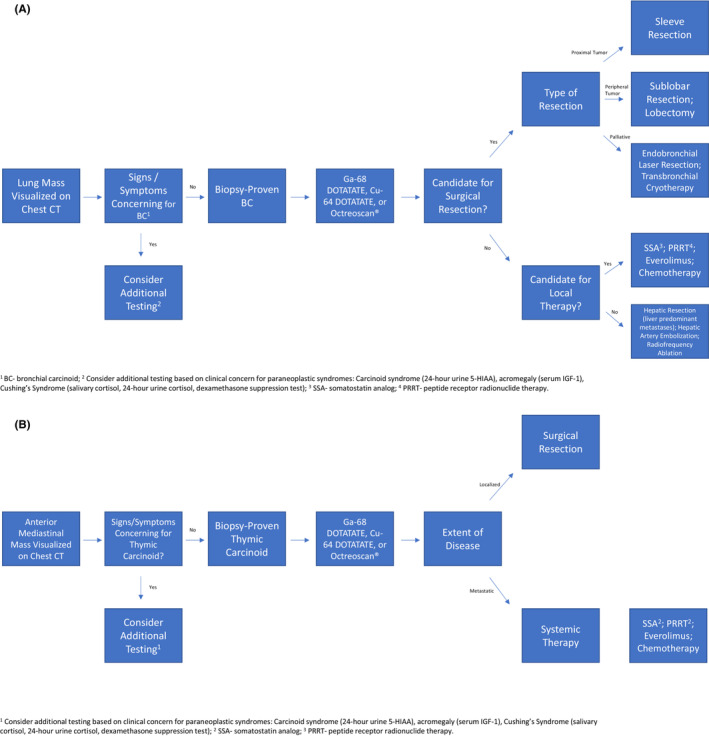
(A) Approach to Bronchial Carcinoid Tumors. ^1^BC‐ bronchial carcinoid; 2 Consider additional testing based on clinical concern for paraneoplastic syndromes: Carcinoid syndrome (24‐hour urine 5‐HIAA), acromegaly (serum IGF‐1), Cushing's Syndrome (salivary cortisol, 24‐hour urine cortisol, dexamethasone suppression test); 3 SSA‐ somatostatin analog; 4 PRRT‐ peptide receptor radionuclide therapy. (B) Approach to Thymic Carcinoid Tumors. ^1^Consider additional testing based on clinical concern for paraneoplastic syndromes: Carcinoid syndrome (24‐hour urine 5‐HIAA), acromegaly (serum IGF‐1), Cushing's Syndrome (salivary cortisol, 24‐hour urine cortisol, dexamethasone suppression test); 2 SSA‐ somatostatin analog; 3 PRRT‐ peptide receptor radionuclide therapy

Complete surgical resection is the treatment of choice in patients with early stage BC (Figure 2A), and it is typically curative for most patients.^10^, ^33^, ^34^ The feasibility of surgical resection is based on size and location of the tumor, comorbid conditions, and patient preference.^35^ Less than 15% of BCs metastasize, therefore there is debate over the necessity of nodal dissection at the time of surgery.^36^ Five‐year survival after resection has been shown to be as high as 90% in patients with localized TCs and 50%–70% in patients with localized ACs.^37^

Sublobar surgical resection involves removing a lung tumor and part of the surrounding tissue in an effort to spare lung parenchyma.^38^ This approach can reduce perioperative morbidity and mortality and preserve lung function compared to lobectomy.^33^, ^35^, ^39^ Given that TCs rarely metastasize or have nodal involvement, these patients are ideal candidates for a sublobar approach. In one study, the sublobar approach was found to be noninferior to lobectomy in patients with TC.^40^, ^41^ When compared to lobectomy, those undergoing a sublobar surgery for localized TC were shown to have similar long term survival compared to those undergoing a larger surgical procedure.^42^ Five‐year survival in patients with TC undergoing definitive surgical management is 90%.^43^, ^44^ Wedge resections are typically not recommended due to studies showing higher recurrence rates and lower survival with this surgical approach.^45^ Surgical management of ACs differs slightly. Many ACs exhibit nodal involvement and metastasis, and some are aggressive, thus necessitating lymphadenectomy for patients with AC, while lymphadenectomy is not required for patients with TC. For patients with thymic tumors, regional lymph node sampling is preferred for patients with AC, whereas similar to among patients with typical bronchial carcinoid, lymphadenectomy can be avoided with the more indolent TC histology.

Endobronchial laser therapy is a palliative local therapy option for patients with intraluminal tumors.^33^, ^35^, ^40^, ^46^ Unless the tumor is completely intraluminal, this approach is less than ideal for curative intent treatment. In most cases, endobronchial laser resection is reserved for central tumors causing symptoms.^47^ In combination with endobronchial laser resection, cryotherapy can also be considered as an adjunct treatment. One study demonstrated that bronchoscopic cryotherapy with Nd‐YAG laser was an effective adjunct to endobronchial laser resection of central carcinoid tumors.^48^ Importantly, cryotherapy is not associated with bronchial stenosis.^48^

There is a lack of data regarding the role of adjuvant radiation or chemotherapy for patients with BC. Patients with TC with clear margins and no nodal involvement at surgical resection have a low risk of recurrence.^49^, ^50^ One retrospective study found that patients with localized TC with nodal involvement who undergo surgical resection did not experience a survival benefit (in fact there was a detrimental effect) with adjuvant chemotherapy.^51^ Additionally, despite a higher rate of recurrence in localized AC with nodal involvement, a large National Cancer Database (NCDB) analysis demonstrated no survival benefit with the use of adjuvant chemotherapy among patients with localized AC with nodal involvement at the time of surgical resection.^49^ Current NCCN guidelines recommend only a consideration (category 2B) of adjuvant chemotherapy in patients with surgically resected AC with mediastinal lymph node involvement.^24^

### 4.2 Thymic carcinoid

The gold standard of thymic carcinoid management is also surgical resection (Figure 2B), which is based on tumor size, location, patient risk factors, and preferences. Complete surgical resection typically involves thymectomy and removal of adjacent anterior mediastinal tissue. The degree of resection affects prognosis and survival.^16^, ^52^, ^53^ Surgical resection can be achieved by transthoracic thymectomy via median sternotomy or a minimally invasive approach. There is a lack of data surrounding postoperative management of these tumors. Radiation therapy with or without cytotoxic chemotherapy is recommended as a consideration in patients with atypical thymic carcinoid tumors with an R1 resection; however, NCCN guidelines note only consideration (category 3) of adjuvant radiation for patients with typical thymic carcinoid with an R1 resection.^54^ Among patients with either typical or atypical thymic carcinoid with an R0 resection, no adjuvant therapy is advised.

## 5 MANAGEMENT OF ADVANCED DISEASE

### 5.1 Bronchial carcinoid

**FIGURE 3:**
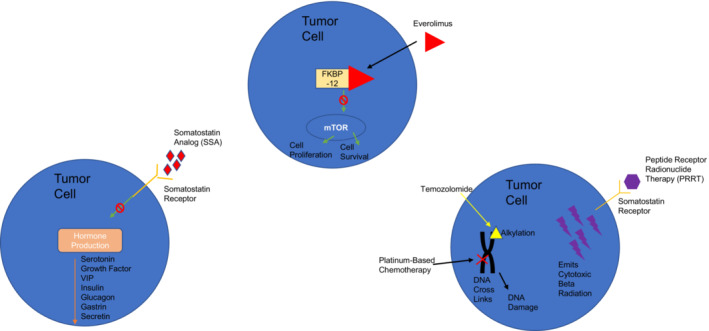
Mechanism of action of current carcinoid tumor systemic treatments

For patients with metastatic BCs or tumors not amenable to resection, treatment involves systemic therapy with a variety of mechanisms of action (Figure 3). Table 2 summarizes the key trials investigating systemic therapy. Overall treatment goals in the metastatic setting are to forestall disease progression, prolong survival, and minimize effects from hormone overproduction.

**TABLE 2:** Seminal systemic therapy trials by treatment mechanism

Treatment	Trial	Patients (#)	Patients with lung or thymic carcinoid tumors	PFS treatment (months)	PFS placebo (months)	OS treatment (months)
Octreotide	PROMID	85	Lung ‐ 0% Thymic ‐ 0%	14.3	6.0	N/A
Lanreotide	CLARINET	204	Lung ‐ 0% Thymic ‐ 0%	Not reached	18.0	No significant difference
Lanreotide + Temozolomide	ATLANT	40	Lung ‐ 90% Thymic ‐ 10%	37.1	N/A	N/A
Lanreotide autogel	SPINET	77	Lung ‐100% Thymic ‐ 0%	16.6	13.6	N/A
^177^Lu‐DOTATATE	Ianiello et al.	34	Lung ‐ 100% Thymic ‐ 0%	20.1	N/A	48.6 for TC 37.0 for AC
Everolimus	RADIANT‐4	302	Lung ‐ 29.8% Thymic ‐ 0%	11.0	3.9	N/A

Abbreviations: AC, atypical carcinoid; ORR, overall survival; PFS, progression free survival; TC, typical carcinoid.

SSAs are drugs that inhibit production of hormones, especially serotonin and vasoactive intestinal peptides, that can be secreted by bronchial carcinoid tumors. Octreotide and lanreotide are considered first‐line SSA therapy in patients with BC tumors expressing SSTR.^30^ SSAs can limit hormone production, slow tumor growth, and stabilize disease in patients with these tumors. Both the PROMID and CLARINET studies demonstrated prolonged time to tumor progression when using long‐acting octreotide and lanreotide, respectively, compared to placebo in patients with midgut and gastroenteropancreatic NETs.^55^, ^56^ However, neither of these studies included patients with BCs. There is retrospective data noting the clinical benefit of SSA monotherapy among patients with metastatic BCs. One study of 61 patients with BCs with functioning metastatic carcinoid tumors showed a median progression‐free survival (PFS) and overall survival (OS) of 17.4 and 58.4 months, respectively, with SSA monotherapy.^57^ Additionally, a retrospective study by Lenotti et al. showed a median PFS of 28.6 months in patients with BCs treated with SSA monotherapy.^58^ The prospective, phase III SPINET trial reported a medial PFS of 21.9 months in patients with TCs and 14.1 months in patients with ACs treated with lanreotide autogel.^59^ Finally, ATLANT is a phase II trial demonstrating safety and efficacy of lanreotide and temozolomide in patients with progressive BCs and thymic carcinoid tumors.^60^

A treatment option for patients with SSTR‐positive BCs at the time of progression or intolerance to SSA is peptide receptor radionuclide therapy (PRRT). PRRT is utilized frequently in patients with GI NETs based on data from the NETTER‐1 trial; however, no patients with BCs were included in this key trial.^61^ There are ongoing prospective clinical trials with PRRT including patients with BCs that we discuss below. Several retrospective studies have reported increased response rates to PRRT with ^177^Lu‐DOTATATE in patients with BCs, with a median PFS between 20 and 28 months and increased survival in TCs and ACs.^62^, ^63^ One retrospective study demonstrated a 5‐year overall survival of 61.4% with ^177^Lu‐DOTATATE versus 31.6% in ^90^Y‐DOTATOC.^64^ The combination of ^177^Lu‐DOTATATE plus ^90^Y‐DOTATOC resulted in an overall 5‐year survival of 61.4%, similar to the ^177^Lu‐DOTATATE monotherapy group, however this combination also demonstrated the highest median OS reported at 61.0 months.^64^

Among patients with carcinoid tumors that do not express SSTR or are not responsive to SSA therapy, treatment with everolimus, an mTOR kinase inhibitor, can be used. Currently, everolimus remains the only FDA approved therapy for patients with metastatic BCs as it is the only systemic therapy with positive phase III randomized trial data (RADIANT‐4).^65^ The RADIANT‐4 trial was a phase III study demonstrating an improvement in PFS and OS, and acceptable tolerability of everolimus compared to placebo in patients with advanced, non‐functioning, metastatic BCs and GI NETs.^65^ Overall, the study enrolled 302 patients with BC or GI NET and reported a median PFS of 11 months versus 3.9 months in the placebo arm. Approximately one third of the patients in RADIANT‐4 had BCs (63 in the everolimus arm and 27 in the placebo arm).^65^ A subgroup analysis of the patients with BC included in the RADIANT‐4 study confirmed the significant improvement in PFS with the use of everolimus compared to placebo (HR 0.50, 95% confidence interval 0.28–0.88).^65^ Seminal trials for each mechanism of systemic therapy used in patients with metastatic or unresectable BC are noted in Table 2.

For patients who have failed the above therapies or have high volume symptomatic disease, treatment with cytotoxic chemotherapy is warranted. Retrospective studies have demonstrated some success with platinum plus etoposide in patients with BC.^66^, ^67^, ^68^ In a single institution, retrospective series among 13 patients with advanced BC (6 with TC and 7 with AC) treated with platinum plus etoposide, 3 (23%) radiographic responses were observed.^68^ Median OS in this small cohort was approximately 11 months, and when including patients with disease stability, the overall disease control rate (DCR) with platinum plus etoposide was 77%.^68^ A second single institution, retrospective study reported an objective response rate (ORR) of 25% with cisplatin plus etoposide among eight patients with metastatic BC.^67^ Data for temozolomide monotherapy is limited, although one study reported a PFS and OS of 5.3 and 23.2 months, respectively, in patients with metastatic BC.^69^ Additionally, there is some data to support capecitabine plus temozolomide or doxorubicin.^70^, ^71^ In a single institution, retrospective series among 20 patients with metastatic BCs (a majority, 70%, with TC) treated with capecitabine plus temozolomide, the ORR was 30% and the DCR was 85%. Median PFS was 13 months, and median OS was 68 months.^70^

The liver is the most common site of metastatic disease from BCs. Patients with limited, liver‐dominated metastases may potentially benefit from surgical resection. Additionally, patients with severe carcinoid syndrome or symptoms of hormone over‐secretion may benefit from hepatic artery embolization and radiofrequency embolization.^72^, ^73^

The brain is an area of infrequent metastasis from carcinoid tumors. One study estimated the rate of brain metastases to be 1.5% in 1,633 patients with any type of low grade neuroendocrine carcinoma.^74^ While the incidence is low, there is increased risk for brain metastases in patients with bronchial carcinoid tumors compared to abdominal, as among the 24 (1.5%) patients found to have brain metastases, 71% of these were due to lung carcinoids.^74^ It is possible that there is an underestimation of the rate of brain metastasis due to lack of routine brain imaging in these patients, as brain MRI is not routinely recommended for well differentiated carcinoid tumors by NCCN guidelines.^75^ Given the overall infrequency of CNS involvement, there is no standard set of guidelines for management, and somatostatin analogs have minimal penetration into the CNS due to the blood–brain barrier.^76^ Decisions on local surgical and radiation options can be individualized through multidisciplinary discussion.

### 5.2 Thymic carcinoid

Systemic therapy for the treatment of patients with thymic carcinoid tumors is similar to treatment used in BCs. SSAs are reasonable treatment options for tumors expressing somatostatin receptors.^77^ Data is limited due to the rarity of these tumors. Temozolomide monotherapy, temozolomide plus sorafenib, and temozolomide plus capecitabine were all found to produce stable disease in a limited number of patients with metastatic thymic carcinoid tumors.^78^, ^79^, ^80^ Poorly differentiated and rapidly progressive disease is typically treated with platinum‐based chemotherapy similar to the management of BCs.^79^, ^81^

### 5.3 Paraneoplastic syndromes

Treatment of Cushing syndrome and acromegaly associated with carcinoid tumors involves reduction of hormone over‐secretion. Surgical removal of the tumor secreting these hormones is preferred. In patients with unresectable tumors, medical therapy for hypercortisolism includes adrenal enzyme inhibitors, such as ketoconazole, metyrapone, and trilostane, or somatostatin analogs.^82^, ^83^, ^84^, ^85^ Intravenous etomidate has also been shown to reduce cortisol levels in patients with severe Cushing syndrome.^86^ Additionally, ACTH secretion from thymic carcinoid has been controlled with combination cisplatin and etoposide.^81^ Somatostatin analogs and dopamine agonists (cabergoline) have been shown to reduce growth hormone levels in patients with acromegaly.^87^, ^88^

## 6 FUTURE DIRECTIONS

Genetic profiling of BCs is an ongoing area of research. In general, studies have shown that the mutation rate is proportional to the degree of aggressiveness of the histology, with more well differentiated tumors (TCs and ACs) having fewer mutations.^89^ One study revealed that *TP53* and *RB1* mutations can be found among all types of bronchial neuroendocrine tumors (TCs, ACs, small cell, and large cell neuroendocrine carcinoma), however their prevalence increases in the more poorly differentiated small cell and large cell neuroendocrine carcinomas.^89^ Additional research into the genomics of BCs may provide further opportunity to improve specific treatments for these patients.

Perhaps most importantly, there are ongoing prospective trials directly comparing mainstays of systemic therapy for metastatic or unresectable BC as well as adding immune checkpoint inhibitor (ICI) or multi‐kinase inhibitors to standards of care for these patients (Table 3). As noted above, most of the therapeutic applications in the treatment of patients with BCs and thymic carcinoid are based on extrapolation of prospective data from patients with GI NETs or retrospective series.

**TABLE 3:** Key ongoing systemic therapy trials for bronchial carcinoid tumors

Treatment(s)	Trial (phase; NCT identifier)	Patients (target enrollment)	Primary endpoint	Secondary endpoints
^177^Lu‐DOTATATE v everolimus	Alliance, phase III, NCT04665739	108	PFS	ORR, OS
Cabozantinib v placebo	CABINET, phase III, NCT03375320	395	PFS	ORR, OS
^177^Lu‐DOTATATE + Nivolumab	HMH008, phase II, NCT04525638	30	ORR	Safety, PFS
Temozolomide + Nivolumab	Phase II, NCT03728361	55	ORR	Safety, PFS, OS
Temozolomide + Cabozantinib	CABOTEM, phase II, NCT04893785	35	ORR	PFS, OS

Abbreviations: ORR, objective response rate; OS, overall survival; PFS, progression free survival.

In a high need evidence space, there is an ongoing phase III randomized clinical trial (RCT) run by the ALLIANCE cooperative group evaluating PRRT with ^177^Lu‐DOTATATE versus everolimus as second line therapy after SSA in patients with BC (NCT04665739). The primary endpoint of this trial is PFS, with a target enrollment of 108 patients and enrollment completion anticipated in 2024. Data from this trial are highly anticipated.

While results with ICIs for the treatment of patients with BCs have thus far been disappointing, including a reported 0% ORR with the combination of nivolumab and ipilimumab in the Dual Anti‐CTLA‐4 and Anti‐PD‐1 Blockade in Rare Tumors (DART) trial among patients with low or intermediate grade non‐pancreatic NETs, there are two ongoing single‐arm trials combining nivolumab with standard of care therapy.^75^ The HMH008 phase II trial is evaluating the combination of PRRT with ^177^Lu‐DOTATATE and nivolumab (NCT04525638) with a primary endpoint of ORR and target enrollment of 30 patients. An additional single arm, phase II trial is evaluating the combination of nivolumab with temozolomide (NCT03728361) in patients with BC, with a target enrollment of 55 patients.

Finally, multi‐kinase inhibitors cabozantinib and surafatinib are under investigation as monotherapy or in combination with temozolomide in prospective trials for patients with metastatic BC. Monotherapy trials of cabozantinib versus placebo (CABINET; NCT03375320), a phase III trial with a target enrollment of 395 patients and estimated trial accrual completion in 2025, and a single arm, phase II trial of surafatinib (NCT04579679), seeking to enroll 76 patients, are ongoing. A single arm, phase II combination trial of cabozantinib and temozolomide (CABOTEM; NCT04893785) is also ongoing.

Particularly the RCTs evaluating PRRT with ^177^Lu‐DOTATATE versus everolimus and carbozantinib versus placebo (CABINET) trial will shed important light on treatment sequencing and prognostic impact of systemic therapy for BCs. Unfortunately, the aforementioned trials do not include patients with thymic carcinoid tumors, whose treatment is anticipated to continue to be informed by data extrapolation and rare, retrospective series.

## 7 CONCLUSION

While BCs and thymic carcinoid tumors are rare, with the addition of PRRT with ^177^Lu‐DOTATATE into clinical practice, there are many interesting and likely practice changing ongoing prospective trials for this patient population. Importantly, these advances are applied in the metastatic or unresectable setting, and definitive surgical resection remains the optimal therapy for patients with BCs and thymic carcinoid tumors. Ultimately, multidisciplinary review of patients with BC and thymic carcinoid tumors in combination with individual patient counseling and partnering in treatment decisions will yield the best outcomes for patients with these tumors.

## AUTHOR CONTRIBUTIONS

**Kenna Koehler:** Data curation (lead); writing – original draft (lead); writing – review and editing (equal). **Wade Iams:** Conceptualization (lead); supervision (lead); writing – original draft (supporting); writing – review and editing (lead).

## FUNDING INFORMATION

None.

## Data Availability

Not applicable.
